# Therapists’ Role in Patient Adherence to Internet-Based Cognitive Behavioral Therapy: Qualitative Study

**DOI:** 10.2196/71852

**Published:** 2025-09-10

**Authors:** Henriikka Anne-Mari Seittu, Tomas Falk, Kushagra Bhatnagar, Suoma Eeva Saarni

**Affiliations:** 1 Department of Public Health, University of Helsinki, PO BOX 20 (Tukholmankatu 8 B), Helsinki, 00014, Finland, 358 505729645; 2 Department of Marketing, School of Business, Aalto University, Espoo, Finland; 3 College of Business, Law and Governance, James Cook University, Townsville, Australia; 4 Department of Psychiatry, Helsinki University Hospital and Helsinki University, Helsinki, Finland; 5 Department of Psychiatry, Faculty of Medicine and Health Technology, Tampere University, Tampere, Finland; 6 Family and Social Services, Psychiatry, Well Being Services County of Päijät-Häme, Lahti, Finland

**Keywords:** internet-based cognitive behavioral therapies, mental health, adherence, qualitative, therapist-assisted internet-based cognitive behavioral therapies

## Abstract

**Background:**

Internet-based cognitive behavioral therapies (iCBTs) are typically categorized into 2 types: therapist-assisted and self-guided. Both formats have accumulated substantial evidence supporting their cost-effectiveness and efficacy in treating a range of mental health conditions. However, therapist-assisted iCBTs tend to show lower dropout rates than self-guided versions. The relatively high dropout rates in self-guided programs suggest that some degree of therapist involvement may be necessary to improve engagement and treatment adherence. Yet, the specific reasons for therapist support in iCBT and its functions in improving engagement and treatment adherence remain an underexplored area of research.

**Objective:**

This study aimed to explore patients’ experiences with therapist-assisted iCBT to identify the elements they perceive as important for treatment adherence and to clarify the role of therapist support in the iCBT process.

**Methods:**

This study draws on 89 semistructured in-depth interviews with iCBT users. Patients took part in 9 different therapist-assisted iCBT programs (depression [n=32], anxiety disorder [n=17], obsessive-compulsive disorder [n=10], bipolar disorder [n=5], social phobia [n=5], bulimia [n=3], alcohol abuse [n=1], panic disorder [n=10], and insomnia [n=6]), all provided nationwide by Helsinki University Hospital in Finland. The interviews were transcribed verbatim and analyzed with the qualitative Gioia method.

**Results:**

Three key categories help explain why users consider therapist support essential for adherence in iCBTs: (1) the strengthening of individual autonomy, (2) the therapist’s commitment to strengthening the therapeutic alliance, and (3) assistance with emotion regulation. Therapist support was shown to be pivotal, often conveyed through small, text-based gestures that had a meaningful impact.

**Conclusions:**

The role of the therapist should not be diminished in the pursuit of digitalization, as human support remains a critical element of effective iCBT.

## Introduction

Therapy services provided by medical experts, such as mental health or physical therapists, represent a vital area where digitalization can have a transformative impact. Previous research has forecasted it as especially effective in tackling resource shortages and enhancing access to therapy [1,2]. For instance, internet-based cognitive behavioral therapies (iCBTs) offer a scalable solution to the aforementioned challenges by providing treatment through web-based platforms or mobile apps. Many studies highlight iCBTs’ cost-effectiveness and their potential to significantly lower mental health expenses [3-6]. iCBTs have demonstrated effectiveness in treating various conditions such as depression, posttraumatic stress disorder, insomnia, anxiety, and eating disorders [7-11]. As their main advantages, they offer flexibility, consistent quality, easy development and implementation, and anonymous treatment options accessible 24/7 [12-15].

iCBTs fall into 2 categories: therapist-assisted and self-guided [12]. In therapist-assisted iCBT, support from the therapist is often remote and asynchronous. While therapist-assisted iCBTs require human resources, self-guided therapies aim to deliver evidence-based treatment to a larger population at a lower cost. However, the challenge of low engagement and significant dropout rates, typically defined as ending the therapy without completing treatment before reaching a predefined cutoff (eg, number of treatment sessions) [16], raises questions about the feasibility of fully digitalized therapy [17,18].

Compared with face-to-face therapies, dropout rates are high in iCBTs [18]. In particular, dropout rates seem greater in self-guided iCBTs compared with therapist-assisted ones, suggesting that some level of therapist support enhances patient adherence. The notion that therapist assistance is crucial for successful therapy outcomes is supported by studies such as Richards and Richardson [18], which found that therapist support reduced dropout rates by 30%‐40%. In addition, therapist-assisted iCBTs have shown greater effectiveness in treating mental disorders compared with their unassisted counterparts [18-21]. An interesting finding, however, was that therapeutic assistance had a greater impact on patients with moderate to severe symptoms, while similar positive effects were observed in patients with mild symptoms even without therapeutic guidance [22]. In other words, it appears that therapeutic guidance is more crucial for those with moderate and severe symptoms than for those with mild symptoms [23].

In sum, therapist support in iCBTs appears crucial for safeguarding adherence and improving treatment outcomes [24-26]. This aligns with findings in traditional psychotherapy literature, which suggest that certain elements in the relationship between therapist and patient, such as alliance or collaborative bond, empathy, and responsiveness to individual client needs, positively affect treatment outcomes, regardless of therapy type [27,28]. However, the link between therapist support in iCBT and adherence is not well understood, especially in terms of why patients find therapist assistance in digital therapy essential and which aspects of therapist involvement are most effective [24,29,30]. To close this gap and to respond to a recent call by Chen et al [31] for more research on the optimal nature of therapist support in iCBT, our study examines patients’ experiences in the Helsinki University Hospital (HUS) therapist-assisted iCBT programs. Based on 89 in-depth interviews, our research aims to identify elements of therapist support that patients consider of critical importance for adherence to iCBTs. The resulting insights allow therapy providers to develop effective strategies to reduce dropout behavior in digital therapy contexts [32-34].

## Methods

### Empirical Setting: HUS Internet-Based Cognitive Therapies

The iCBT program under investigation is a nationwide service provided by the iCBT clinic at HUS Psychiatry. These HUS-iCBT programs are diagnosis-specific, therapist-supported, and free of charge for patients. To participate in the HUS-iCBT program, a referral from a physician is required. HUS-iCBT programs are part of the public health care services and can be accessed from anywhere in Finland, regardless of location.

The HUS-iCBT program is tailored for individuals experiencing mild to moderate mental health challenges. While all physicians in Finland can refer patients to HUS-iCBT, enrollment requires a physician’s referral. At present, 12 different programs are offered, addressing conditions such as depression, social phobia, panic disorder, generalized anxiety disorder, attention-deficit/hyperactivity disorder, alcohol abuse, insomnia, obsessive-compulsive disorder (OCD), bulimia nervosa, long-term physical symptoms, and mental health support for patients with cancer.

Each HUS-iCBT program lasts approximately 8-12 weeks. Patients participate in weekly sessions that combine educational content related to their diagnosis with written assignments and diary entries. After an initial phone call, therapist support is provided asynchronously via written messages, offering encouragement, answers to questions, feedback on assignments, and responses to symptom questionnaires. Additional phone calls or text messages are arranged if necessary, particularly if a patient shows limited progress in therapy or exhibits self-destructive behaviors. The therapists involved in the program include clinical psychologists, psychology students, and psychiatric nurses with additional therapeutic training. All therapists receive dedicated training and clinical supervision to ensure the quality of care.

### Data Collection

The resulting dataset comprised 89 in-depth interviews with former patients who had received iCBT at HUS. Patients took part in 9 different therapist-assisted iCBT programs (depression [n=32], anxiety disorder [n=17], OCD [n=10], bipolar disorder [n=5], social phobia [n=5], bulimia [n=3], alcohol abuse [n=1], panic disorder [n=10], and insomnia [n=6]). To solicit interviews, inquiries were distributed through HUS’s email list to all HUS-iCBT users who had undergone the treatment in the past 6 months. The inclusion criteria for the recruitment were being of legal age and having participated in one of the HUS-iCBT programs within the past 6 months.

Conducted in spring 2022, the majority of interviews (87/89) were carried out remotely via platforms such as Zoom (Zoom Video Communications), Teams (Microsoft), phone, or Skype (Skype Communications), primarily due to COVID-19–related restrictions and the geographical dispersion of participants. All interviews were conducted by the first author. To ensure clarity and comprehensiveness, the interview guide was piloted and subsequently refined before data collection. Data collection and analysis were conducted concurrently, allowing for iterative assessment of data saturation throughout the coding process. Recruitment of participants was stopped when no substantially new codes, themes, or insights emerged from additional interviews, indicating that thematic saturation had been reached. This determination was further validated through regular team discussions, during which the completeness and depth of the emerging themes were critically evaluated.

All interviews—ranging in length from 21 to 134 minutes, totaling 4136 minutes—were audio-recorded and transcribed verbatim, yielding 1154 pages of single-spaced text (Times New Roman, 12-point font). The interviews focused on key themes, including participants’ overall experiences with and expectations of iCBT, the referral process, the perceived role and value of therapist support, patient needs during treatment, perceived benefits and limitations of iCBT, the practical demands and competencies involved in its delivery, and the role of technology in therapeutic work. A detailed interview guide is available from the corresponding author upon reasonable request.

### Data Analysis

**Figure 1.**
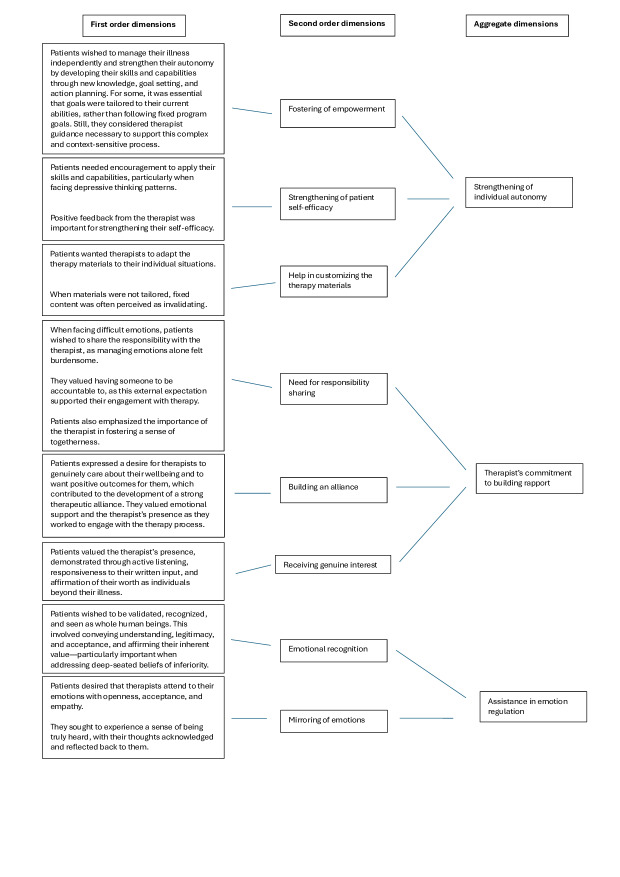
Data structure.

To better understand patients’ needs for therapist assistance, this study adopted the Gioia method [35], an inductive research method for generating grounded theories in data analysis [36]. The method was chosen to identify concepts, illustrate their interrelationships, and describe a specific phenomenon—in this case, critical facets of medical expert involvement in iCBTs that shape patient adherence. Following the Gioia method [36], the data underwent coding and analysis, resulting in a coding structure presented in Figure 1. A structured presentation of data was chosen as Gioia et al [36] argued that it is essential for effectively conveying the authors’ knowledge to the reader.

The Gioia method operates on the assumption that informants are “knowledgeable agents” capable of providing information on their emotions, actions, and thoughts. The method involves classifying collected data using the language used by informants, which is then transformed into abstract, researcher-driven themes that describe the phenomenon from a scientific perspective.

The analysis process began with open coding of transcripts line by line to derive meaning from the data and uncover significant insights. This involved identifying patterns, themes, and concepts central to the research question. In this part of the coding process, Atlas.ti (ATLAS.ti Scientific Software Development GmbH) software was used to mark and classify the codes. These codes were informant-centered, presenting the informants’ experiences in their own terms and language [36]. Notes and descriptive comments were added to the transcripts during this phase, and memos were written to summarize the main points of the interviews, managing the volume of transcriptions under research. Memos served as summaries, focusing on the most important content of the interview.

Following open coding, first-order dimensions were drawn directly from the raw interview material. These first-order codes were then clustered into conceptually similar groups, forming second-order themes that were theory-centered. In this phase of coding, scientific understanding of the phenomenon guided the terminology used to define the themes. The purpose of constructing second-order themes was to identify relational patterns and underlying dimensions within the first-order codes [36]. In this study, second-order themes captured participants’ needs and preferences regarding therapist assistance. Throughout the coding process, special attention was given to identifying and incorporating negative or contradictory themes, ensuring that divergent or opposing participant perspectives were represented and critically examined. Finally, aggregate (third-order) dimensions were developed to represent the most abstract and overarching concepts emerging from the data. Overall, 8 second-order themes and 3 aggregate dimensions were identified.

To illustrate the process, first-order concepts were derived from direct patient quotes. Quotes such as the 2 below highlighted how patients sought to manage their illness independently but initially required therapist support for building confidence, tailoring tasks to their capabilities, and overcoming emotional barriers.

*This fosters a sense of empowerment because you realize you can do it on your own and continue to apply these skills in future situations*.

[Anna, 44]

*You can’t do those exercises alone, but when the therapist was there in the background, I could do them*.

[Sally, 54]

These quotes were grouped under the second-order theme “fostering of empowerment.” This theme reflects how therapist assistance promoted patients’ ability to take control of their therapy and symptom management. The second-order theme was further integrated into the aggregate dimension “strengthening of individual autonomy,” representing the broader goal of enhancing patients’ agency in managing their conditions.

As already indicated, the first author was primarily responsible for conducting the interviews and leading the entire analysis process, including coding and theme development. This work was conducted in close consultation with coauthors who contributed complementary expertise in mental health care and service research. The first author’s multidisciplinary background in health care, service management, and social sciences, combined with substantial qualitative research experience, enabled her to approach the data from diverse perspectives, helping to mitigate potential biases.

Interrater reliability was addressed through an iterative dialogue process within the research team. The first author developed initial coding proposals that were systematically and critically reviewed in regular collaborative meetings with senior coauthors. This process typically involved revisiting the original data, jointly re-examining transcripts, and negotiating interpretations until consensus was reached. These collaborative discussions fostered continuous critical reflection, during which preliminary findings were scrutinized, divergent views debated, and coding frameworks refined. This collaborative process helped prevent overreliance on the first author’s interpretations alone, ensuring shared understanding and reducing potential bias.

In cases of disagreement or conflict regarding coding or interpretations, differing perspectives were documented in the audit trail. In this context, the audit trail refers to a transparent record of analytical decisions and reflections made throughout the research process, allowing others to trace how interpretations and conclusions were reached [37]. A comprehensive audit trail was maintained throughout, documenting every step—from refinement of the interview guide and reflection on preliminary interpretations to development of analytical themes, coding decisions, and selection of representative quotes. The audit trail also recorded reflections and resolutions of disagreements among researchers. This thorough documentation enhanced transparency and allowed coauthors to critically review and validate the analytical steps, thereby strengthening the trustworthiness and credibility of the study.

### Ethical Considerations

This study was approved by the HUS Committee on Medical Research Ethics (HUS/821/2021). Patients provided informed consent before participating in the interviews. No compensation was provided to the study participants. In the transcription and data analysis process, pseudonyms were used to replace all the personally identifiable information, such as names and disorders, ensuring anonymity. Because of the highly private nature of the study participants, only name, age, HUS-iCBT treatment program, and living region were collected as patient information. Only these data were collected because collecting as little personal information as possible was a requirement of the ethics committee. To ensure data security, the original recordings were deleted after transcription. The transcribed data were stored as password-protected files on secure servers of the research organization, with access limited to the research team only.

## Results

### Overview

The sample incorporated 60 female and 29 male participants, aged between 19 and 76 years, residing in various regions across Finland. Notably, almost all patients expressed a desire for increased therapist assistance, such as through phone calls or online meetings. It is also noteworthy that patients in this study compared iCBTs with their idea of traditional face-to-face approaches, despite having limited experience with the latter. To illustrate these experiences—particularly those related to the strengthening of individual autonomy, the therapist’s commitment to building therapeutic alliance, and assistance in emotion regulation—specific examples are presented below.

### Strengthening of Individual Autonomy

#### Fostering of Empowerment

Empowerment emerged as one of the patients’ most sought-after outcomes of therapy. However, achieving empowerment was perceived as challenging when undertaken alone, requiring external support from the therapist. The data frequently emphasized the importance of a therapist’s initial guidance in building confidence and empowering patients to take control of their treatment. It also revealed a clear need for therapist assistance in tailoring knowledge and skill-building to align with patients’ current capabilities. Patients underscored the therapist’s importance in initiating individualized support, ensuring that patients feel capable of managing their conditions on their own, but also assessing illness-related responsibilities based on each individual’s unique, disorder-specific circumstances.

*From my experience with iCBT, I learned that I could influence my symptoms myself. The iCBT involves working step-by-step with the therapist to perform various exercises. This process gave me the feeling that I was doing the exercises on my own. When you start by doing the exercises with the therapist’s support, it eventually feels like you are guiding yourself through them. This fosters a sense of empowerment because you realize you can do it on your own and continue to apply these skills in future situations. You get support to utilize your own abilities, and once you begin doing the exercises alone, it feels natural to continue doing so. This approach is always beneficial, but I believe it is crucial to understand the individual’s situation and assess future responsibilities accordingly.*

[Anna, 44]

As becomes apparent in Anna’s case, it was crucial that the patient’s situation was understood and goals were aligned with her skills and capabilities. That was also the experience of many other patients. For instance, Saga, a 20-year-old woman with depression, found basic tasks such as eating and showering challenging. She required the tasks presented in the iCBT materials to be adjusted to better align with her capabilities to effectively commit to those activities.

In some cases, helpful therapist assistance meant pushing the patient forward to reach the goals when adherence with iCBT felt difficult for the patient for one reason or another. This was the case for Sally, a 54-year-old woman with OCD, who, in the face of negative emotions and chaos, describes how she managed to comply with challenging assignments only when the therapist pushed her forward.

*These obsessions are a really difficult thing, they sound completely irrational, but it’s a terrible state when they’re active. I told her how awful it is, and it felt like she understands what it feels like, that she understands how bad this is, but we don’t just lie down in this fire, we move forward. ... It was precise and factual, but she really delved into these things. It was, there was no nonsense. I don’t like that. I like that there’s substance, and it just felt like she was talking specifically to me ... When I had something difficult, she had read what I had written and just felt that she understood what kind of difficulty I had experienced. She stood there beside me and said, “Yes, this is, I know, but we continue here.” You can’t do those exposure exercises alone. It always stays like this, but when the therapist was there in the background, I could do them. It’s really important that the person is there on the other side of the internet.*

[Sally, 54]

In her narrative, she emphasizes how the therapist’s presence was crucial because she felt the therapist understood her situation and spoke directly to her by delving into things she mentioned as challenging, giving her the feeling that she was not alone. This is again something for which a therapist is needed to create a personal connection with all its emotional nuances and relatability and to sensitively monitor the patient’s personal situation and offer real-time adaptation.

#### Strengthening of Patient Self-Efficacy

Strengthening of patient self-efficacy was another element frequently highlighted in the patients’ narratives. Strengthening patient self-efficacy was especially crucial for patients because, as Saga, a 20-year-old woman with depression, explains, with mental health problems, even small things can feel insurmountable, and there is often a tendency to think negatively. Therefore, it is especially important to praise activities that promote recovery. Without praise, Saga explains, they may sink deeper into the despair of depression if there is no entity that acknowledges the small yet significant efforts from the perspective of someone dealing with the illness.

*If you just manage to do some small hygiene thing or make food for yourself, it would be good if there were a message like, “Well done, you were able to do it.*” *In a way, even praising that if you can’t do all the requested things but manage to do a small act, like brushing your teeth once a day, even if you can’t do anything else, someone reminds you that it’s enough. You’re praised and encouraged for that effort. Otherwise, if it had been structured like, remember to maintain hygiene today, and then I couldn’t do it, it might have made me feel a bit bad, like oh, I can’t do that either ... That encouragement and praise are important because in depression, there is a tendency to think negatively about everything.*

[Saga, 20]

In Saga’s experience, she found that having support for her self-efficacy was crucial for maintaining adherence. Genuine and emotionally involved praise and empathy are human expressions that carry emotional weight and cannot be replicated by a self-guided iCBT. Patients reported that the best support came from another human who could truly understand the nuances of a patient’s current emotions and adapt their response accordingly. However, the findings also show that automatic messages can create such feelings if the message creates an authentic experience for the patient.

#### Help in Customizing the Therapy Materials

The need for patient enablement was actualized as help in customizing the iCBT materials. In the data, customization appeared as help with modifying the fixed material to the patient’s personal situation. Examples of modification were giving tips to apply the given examples to the patient’s situation or asking the patient to focus on something special and important for his or her situation in the materials.

*Perhaps now, when I think about it, it would have been nice, maybe good to get a kind of toolbox tailored to my own needs, a workbook to take along. If you could have received something like that, saying, “These are now things that you should focus on, you have this area that’s like this, so these exercises would suit you, do them like this, and stop when it feels good.” Maybe something like that would have been good, something to take away. Like, when [laughs] I play golf, I once went to a golf instructor who wrote on a small note,* “*Pay attention to this and this thing.*” *Every time there were problems in the game, I would then look at that note and go, “Oh right, that and that thing,” so maybe something like that is what I mean, that there would be a toolbox to take along. So that it’s like, what’s your thing, what you need to focus on.*

[Matt, 34]

Like Matt’s quote illustrates, he implies that therapist assistance was crucial because the therapist can provide tailored advice and exercises suited to his specific symptoms and challenges. Again, the therapist’s ability to provide real-time and personalized monitoring of the patient’s situation becomes crucial. In addition, the therapist can ensure that the patient receives precise and relevant guidance, helping them understand how to proceed with his or her personal iCBT journey. This level of personalization is difficult to achieve through self-guided iCBTs alone because they cannot sense nuances, such as emotions linked to the patient’s perception of their capability to complete iCBT tasks.

The need for customized materials was also relevant for Polina, a 23-year-old woman with an anxiety disorder. Her quote emphasizes the importance of tailoring iCBT content to an individual’s unique circumstances. With the therapist’s guidance, she gained clarity on how to apply the iCBT techniques in a way that suited her specific situation.

*I didn’t really understand how this example or this technique or this exercise fits my situation, because I think some of the examples used in the online program were quite banal or superficial or … It was hard for me to make connections between them and my own thoughts. But my therapist helped me to understand how I can use the same technique in my situation. … We were able to highlight the pain points and find the important themes which we should examine closer. So that sort of thing would be completely missed out on only a self-guided iCBT program. And that helped me to find new paths that I hadn’t even regarded as reasons for my bad feelings, so they were able to find that sort of stuff, my pain points. … Yeah, and the individuality, because only such an online program can never know my personal situation, so another human is needed. Although there are some universal things that pertain to several people, but it is hard for me to see that such iCBT could be as individualized, if there is no real person on the other end.*

[Polina, 23]

Polina contends that achieving individualization would have been challenging without interaction with the therapist. Indeed, if patients do not perceive the general iCBT materials as relevant to their specific situation, therapist support to adjust the materials might be crucial to improving adherence. Similar was also the case of Saga’s:

*In the iCBT, it didn’t really take into account how difficult it can actually be when sometimes you just can’t, I mean, can’t even go to the store for a week or can’t muster the energy to do the dishes or something. In depression, making even a simple meal can be a significant challenge, and in the materials, it was just assumed that a person can cook for themselves, even though the illness makes it really difficult. So, it would have been more beneficial if the therapist acknowledged that, yes, this might be a big step, but take it one small step at a time. Like, first, make sure you have food in the pantry. If you don’t, go to the store. So, gradually go through the implementation of that aspect.*

[Saga, 20]

Like Saga explains, to adhere to iCBT, the goals needed to be adapted to the patient’s personal situation. Therapist assistance was preferable to self-guided iCBTs, as the patient’s situation needed to be monitored throughout the iCBT process, since the capabilities of the patient might vary from time to time. The therapist was perceived to be able to monitor the patient’s personal situation and its nuances better than a self-guided iCBT, which is logical because, compared with a self-guided program, only the therapist could make complex, context-sensitive, and real-time judgments about a patient’s condition and target tasks accordingly.

### Therapist’s Commitment to Strengthening the Therapeutic Alliance

#### Desire for Responsibility Sharing

The first aspect of strengthening the therapeutic alliance is the desire for responsibility sharing, which presents an intriguing finding, as it may seem contradictory to the desire for individual enablement, where patients seek independence. However, these preferences should not be viewed as mutually exclusive, as patients’ wishes are often diverse and, at times, conflicting. For certain aspects, such as symptom management, patients prefer working independently, while for dealing with emotions and experiences, they may wish to share the responsibility with the therapist. Saga, for instance, highlights her feeling of accountability to the internet therapist, emphasizing how this presence assisted her in adhering to iCBT, as she knew someone was waiting for her to log in.

*When there are two people involved, the other person also ensures that—In a way, when you are alone in online therapy, it feels like it’s solely on your shoulders, but when you’re with someone, it feels like, well, this therapy is the responsibility of both of us. It’s like you feel compelled to log in a bit for the sake of the other person.*

[Saga, 20]

#### Building a Therapeutic Alliance

The second dimension of the therapist’s commitment was the need to build an alliance. This alliance, formed between the therapist and the patient, was frequently highlighted in the data as a key factor in helping many patients adhere to iCBT. For example, Daisy, a 60-year-old woman with an anxiety disorder, emphasizes her desire for a therapeutic alliance and the therapist’s genuine concern for her well-being—a sentiment shared by many patients. She underscores the importance of having someone who cares for her, assesses her situation, and shares responsibility to ensure compliance with iCBT assignments. As Daisy explains:

*So, there is no, so that sort of thing and if the situation changes, it should be assessed. So, there should always be someone looking after you, because you wouldn’t realize, if there’s a change in your situation – because you are quite alone in there… That your self-care is constantly supported.*

[Daisy, 60]

Daisy’s account highlights the sense of loneliness that patients often report feeling when working alone in iCBT. The expectation of having another person available in iCBT significantly influences patients’ perceptions. In some cases, patients mentioned that the therapist did not necessarily need to take specific actions but should be available if needed. Harry, a 30-year-old man, sums it up:

*A personal and empathetic response increased my attitude towards the service, making me feel like this is for me, and that the person there is also for me. I always had the impression that I wasn’t just filling out a survey, but rather, I felt that the person was there for me.*

[Harry, 30]

Furthermore, alongside building an alliance, interviewees expressed a desire for the therapist to truly care about achieving positive outcomes for them. Lauren, a 42-year-old woman with anxiety disorder, illustrates this by highlighting the importance of the therapist’s genuine desire for her well-being:

*For me, it has been extremely important that to my experience, the health care professional is on my side when it comes to those matters. That they really want good things happen to me. … To have the chance to tell you have a need. And that it’s accepted, and that it’s not downplayed, and that it’s not - it’s just that you have a need, and that’s all there is to it.*

[Lauren, 42]

Lauren’s account underscores the necessity of having a therapist who not only listens but also truly cares about the patient’s positive outcomes. This level of empathy and emotional investment is something that therapists are taught in their education and, for example, peers might lack. Often, the therapist is sought after by many patients because not everyone has a peer or relative who shares the same level of interest in their positive outcomes.

Although many patients found it possible to build a therapeutic alliance in iCBT, not all reported positive experiences, as exemplified by Hannah, a 36-year-old woman with depression. She offers a detailed account of her expectations for building a therapeutic alliance in iCBT and the aspects that left her disappointed, particularly due to the therapist’s generic responses:

*The messages were quite generic, like, “Hey, good for you, you’ve progressed nicely and stuff.” It was broadly encouraging, you know, like, “Nice, thanks [laughs].” The therapist could have picked something from there, like, “Hey, did you notice that this could be approached from this or that perspective?” So, in a way, I feel it went a lot like I was just being affirmed, like, “Yeah, that’s right, that’s how it is.” It didn’t really open up new perspectives for me; instead, it was more like constant agreement, like, “Yeah, that’s how it is.” … It might have been beneficial if there was someone who would grasp onto some of my thoughts and advance those issues through that. For me personally, it’s like I get a reflective surface for my thoughts and emotions. I’m the kind of person who can be told quite directly, like, “Have you noticed that there’s a lot of inconsistency here or something?”… It’s like I get a kind of reflective surface for my thoughts and emotions … Those copy-paste responses, well, they don’t really move the issue forward much.*

[Hannah, 36]

Furthermore, the need for personal contact is further emphasized by Jason, a 26-year-old man with depression, who points out that the therapist’s presence plays a crucial role in fostering a sense of togetherness and preventing feelings of isolation. The therapist’s ability to build a trusting alliance, understand and validate the patient’s needs, and provide consistent, empathetic support is integral to the therapeutic process. This personal connection and genuine concern cannot be adequately replaced by a peer or a self-guided iCBT, as they lack the professional training, objective perspective, and emotional depth required to meet the patient’s psychological needs. Jason succinctly captures the prevailing sentiment in his narrative:

*The [personal contact] gave me a feeling that I’m genuinely important to him, to that therapist. Knowing that they know who I am, so they can talk to me about my issues.*

[Jason, 26]

Some patients rationalized that the need for a therapist is particularly crucial because, in mental health problems such as depression, the feeling of loneliness is prevalent. In many cases, indeed, patients mentioned that they had no one else to talk to about their situation, making the therapist the only person aware of their circumstances. Sharing emotions or being present is something that a self-guided iCBT cannot deliver. As David, a 34-year-old man with depression, explains:

The most important thing, in my opinion, is that a person doesn’t experience being left alone and that they have a tremendous amount of responsibilities because, as someone with depression, you might not be able to carry that burden alone.

[David, 34]

#### Receiving Genuine Interest

Finally, building a therapeutic alliance was crucial when the therapist showed genuine interest in the individual rather than just focusing on the disorder. Anett, a 28-year-old woman with bulimia, emphasizes:

She commented that when he asked me how I was doing, and I wrote about it, in the next message, she reacted to those things I wrote about and might ask about them, like how did this go. It conveys that he is interested in me, not just, you know, he is now forced to write me a message once a week. ... Personally, I specifically longed for a listener and someone who is interested in you. Because those people who can endure that you are broken and can be present with it, accept it, and be present, are quite rare. So, I longed more for that presence, that someone, for once, asked how you are, focused on your story, not so much advice.

[Anett, 28]

As Anett’s account illustrates, both the therapist’s presence and acceptance were crucial elements. The presence of a therapist offers a sense of companionship and validation. This communication with the therapist is vital for patients to feel understood and supported, as Anett’s case shows. Another account highlighting genuine interest, encompassing various aspects of the other elements, comes from David:

*But yes, the therapist’s presence was really important so that I wasn’t just rambling there by myself, but there was someone who heard and listened, discussed those things, and was the professional on the other end of the line. If I felt down, they encouraged me at that point, urged me to share more, and if things were going well, they were there to say “hey, great job” and “tell me more thoughts if they come to mind.” They genuinely, [laughs], said it, but they genuinely read and thought about the texts I had sent there and the results I had obtained, not just from those mood charts or not charts but from when I assessed my own mood and coping. Immediate feedback was given on those issues, and that was important for my well-being.*

[David, 34]

### Assistance in Emotion Regulation

#### Emotional Recognition

In emotional recognition, patients felt that their emotions were validated and, as they describe it, they were “seen as a human being.” Due to their condition, many patients were insecure, had limited self-validation skills, and low self-esteem, making recognition from the therapist crucial. For instance, Rebecca, a 45-year-old woman with social phobia, describes how emotional recognition—the experience of being seen—was the element that enabled her recovery:

*It means that you come to be seen, and in a way, it provides an opportunity for healing. In that moment, you feel that you can heal and acquire good emotional tools. Being seen is a significant, almost invaluable thing. Not almost, but it really is. At least for me, it’s such a huge weight lifted off my heart.*

[Rebecca, 45]

An additional aspect of emotion recognition highlighted the significance of validation. The concept of validation recurred frequently in the data, exemplified by Marie, a 32-year-old woman with depression. Marie elaborates on how validation could stem from affirming the value of the patient, particularly someone who feels unworthy. Even if subconsciously, she believed that this validation contributed to her recovery and adherence to the treatment. She describes:

*Validation is important. It should be present even if the person doesn’t accept it. ... It is the fundamental, perhaps assumed, aspect for individuals that makes things challenging, that I am somehow wrong because I have these feelings or I am somehow different, or I am somehow inferior to others. So, getting rid of that thought right away is crucial to even initiate the process of healing and therapy, because it is a completely false notion that anyone is better than another, regardless of what is happening in their lives. But on an emotional level, it may still be true for that person, even if logically and rationally, it is just as you say, and you accept that fact. But on an emotional level, it may not be true at all for that person, so constant validation is needed - just to confirm or reinforce that thought [that one is not inferior to others]… Even if it is not accepted, it still needs to be there because it may subconsciously help in some way. External validation or affirmation is necessary.*

[Marie, 32]

The quote illustrates the importance of validation in iCBT and underscores why only a therapist can provide this effectively. Therapists offer empathy, authenticity, and a genuine connection, addressing both the conscious and subconscious emotional needs of patients. This validation is crucial for challenging and reframing deep-seated beliefs of inferiority, fostering a sense of worth, and initiating the healing process.

The need for emotional recognition was also crucial in Julia’s case, a 32-year-old woman with panic disorder. She expressed a desire for more emotional acknowledgment, feeling that in iCBT, discussions were limited to practical matters. Julia emphasized the importance of the therapist comprehending her situation and the challenges she faced. She highlighted the significance of acceptance and the therapist’s presence in fostering a sense of security. However, she noted that acceptance and presence were somewhat contingent on the effectiveness of the therapeutic alliance. In her situation, dissatisfaction with the therapist’s support and the lack of a strong therapeutic alliance made it challenging for her to adhere to the iCBT:

*In them, there was a lot of encouraging feedback and they were indeed friendly, but perhaps what was lacking was a sort of understanding of what you are going through or an attempt to understand what made it different was that I didn’t perceive it as therapy because there was no therapeutic connection with the person; they were more focused on practical matters that could be discussed.... The establishment of trust requires a sense of being seen and understood and how it creates that feeling of security around those matters, of course, it also depends on the chemistry, but it’s a kind of acceptance and allowance, like presence.*

[Julia, 32]

Like Julia’s case shows, talking about practical matters was not enough for the patient to feel heard and understood, which was regarded as important. For the therapeutic connection to be established, the feedback Julia received needed a deeper understanding of her emotional state and experience. This kind of nuanced recognition requires empathy and the ability to relate to the patient’s feelings. Again, as becomes apparent, personalization and the feeling of being seen and heard are central in understanding patients’ unique challenges and emotional struggles. Another example of feeling truly understood comes from Justin, a 46-year-old man with panic disorder, who elaborates on his interaction with the therapist:

Another example of feeling truly understood comes from Justin, a 46-year-old man with panic disorder, who elaborates on his interaction with the therapist:

*Also, the fact that the therapist knows how to ask those right questions, and through that, it conveys a genuine interest in understanding how the patient is doing and coping. It also reflects a sincere willingness to help, finding the tools together to move forward. Of course, it’s good for the patient to know that the therapist is not the one who solves the problems; one must do the work themselves. The therapist is specifically the one who poses the right kind of questions, guiding and providing food for thought.*

[Justin, 46]

As becomes apparent, Justin believes that “asking the right questions” is the therapist’s responsibility. This can be explained by the therapist’s ability to convey a genuine and sincere willingness to help the patient, as well as to understand the nuances of human emotions and tailor their questions accordingly—something that purely self-guided iCBT cannot fully achieve. Indeed, every patient emphasized that a predetermined, self-guided iCBT lacked the ability to deliver, receive, or effectively address emotions.

#### Mirroring of Emotions

The second aspect of emotion regulation involved the mirroring of emotions. For the patients, this entailed that the therapist openly, acceptingly, and empathetically received the patient’s emotions. The help of the therapist was needed in this, for example, because the therapist’s reception of the emotions conveyed the experience of emotional validation to the patients. For example, engaging with the emotions of the patients and mirroring those emotions back were vital aspects that the patients felt they could not deliver themselves without the help of the therapist. Two quotes below illustrate patients’ reflections on the reception of their emotions in iCBT:

*I’d say the starting point is that the customer gets heard. It’s also essential that the customer feels that they’re accepted, no matter how bad their situation is or how crazy things they do or how weird their thinking is or how stuck they were with their own problems… It’s some sort of a reflective sentence. Even though it can theoretically be that the caring person just repeats something I have said, but it’s intuitive to some extent, the experience of being heard – it was crucial for me and with it I could start the therapy.*

[Leila, 63]

*It’s down to the fact that your emotions and thoughts get reflected back. You’ll get the feeling of being safe, the basic trust. Only that shuts down your flight-or-fight mode and you can think clearly.*

[Minnie, 19]

They both emphasize the significance of being heard, particularly through reflective responses infused with acceptance. As in Leila’s case, the feeling of being heard was the initial starting point for her to start the iCBT. A similar sentiment is echoed by Minnie, a 19-year-old woman dealing with panic disorder. She elaborates on how the act of hearing and reflecting thoughts and emotions back to her played a pivotal role in establishing fundamental trust. This made it possible to deactivate the fight-or-flight mode, which is crucial for gaining the reflective position needed for therapeutic work. For Minnie, cultivating this foundational trust was essential for achieving mental clarity and, consequently, effectively engaging in iCBT tasks.

Expanding on previous accounts, Jenny, a 25-year-old woman with depression, elucidates how being heard played a pivotal role in kickstarting her recovery journey, granting herself permission to rest. She articulates that before experiencing being heard, she struggled with the belief that she lacked the permission or rationale to pause and initiate the recovery process. Furthermore, she contends that the absence of the therapist at the other end of iCBT would have left her feeling profoundly distressed. She explains:

*I just wished that I could be, but then I guess I also hoped that someone would have heard me and taken a part of that burden off me. Something different than being told that I should change my behavior myself, especially when that was the very thing I couldn’t do. … Just the fact that I was heard was a significant part of being able to start recovering or allowing myself to rest. Until then, I just couldn’t do it because I felt like I didn’t have a reason or permission. It would have been terrible if there hadn’t been another person at the other end. Or if it had felt like this was just some kind of self-paced correspondence course, that there wasn’t enough of another person for you. Because the proximity of another person is part of basic needs, and especially now during the time of COVID-19, one has noticed its significance.*

[Jenny, 25]

Finally, a crucial aspect of emotion reception involved paying attention to the patient’s emotions. For the patients, this was significant because they desired to feel valued by the therapist and to be understood in therapy. Fanny, a 60-year-old woman with social phobia, emphasized the importance of attending to her emotions and expressed that this connection could be established through written communication:

*The important thing was the therapist’s participation in my emotions, so if I told them that something had made me really happy, their response would reflect that they had read and engaged with my positive feelings. The use of words is very meaningful; while in face-to-face sessions one might observe someone’s expressions, in this context, they probably focus more closely on the words.*

[Fanny, 60]

## Discussion

### Principal Findings

The findings of this study underscore 8 key aspects that patients receiving iCBT deem crucial for their engagement in internet-based therapeutic treatment—empowerment, strengthening self-efficacy, customizing of therapy materials, responsibility sharing, therapeutic alliance, receiving genuine interest, emotional recognition, and mirroring of emotions. In alignment with arguments made by Baumeister et al [20] regarding the superiority of therapist-assisted iCBTs over purely self-guided ones, this study offers a detailed explanation for why therapist assistance is perceived as vital for safeguarding patient adherence. Our findings extend previous knowledge by providing an in-depth understanding of why the presence of a therapist is crucial, positively contributing to patient satisfaction and adherence in iCBT.

This study guides on identifying which attributes should not be digitalized and which aspects could potentially be replicated by technology to reduce the therapist’s involvement. Overall, iCBT users clearly expect certain elements to be delivered by the therapist, and a purely self-guided version may be perceived as inferior. For example, a therapist’s presence ensures that patients do not feel alone in their journey, offering emotional support, validation, and dynamic interaction—qualities that automated programs cannot replicate. This therapist’s assistance is essential for patients to feel understood, supported, and motivated. Elements such as accountability, presence, therapeutic alliance, personalized encouragement, real-time adaptation, validation, emotional connection, and relatability are all aspects that purely self-guided therapies cannot deliver. In addition, trust in a therapist’s expertise, as emphasized in many patients’ accounts, is something that self-guided iCBTs cannot yet provide. These insights, which highlight the importance of the therapist’s role in iCBTs, align with the findings of Pearcy et al [19], who identified a positive relationship between OCD outcomes and the number of therapeutic hours. Overall, the implications of this study underscore the need for a balanced integration of therapist involvement and self-guided digital materials to ensure cost-effectiveness, positive well-being outcomes, and patient satisfaction. The most suitable combination should be tailored to the patient’s needs.

It is noteworthy that most of the themes highlighted by patients in this study—such as therapeutic alliance, empathy, and responsiveness to individual needs—are common concerns in psychotherapy in general [27,28]. In other words, on the one hand, the very same elements seem to strengthen the therapeutic alliance and adherence in psychotherapy, independently of the mode of delivery. On the other hand, the iCBT-specific aspects found in this study highlight a few important considerations. In iCBT, the feeling of being seen and heard, validated, and supported seems to come from small text-based gestures. The therapist must make complex, context-sensitive judgments about a patient’s condition through written communication alone, making the therapist’s reading and writing skills central in a way that differs from traditional therapy. Furthermore, the patient’s writing skills are also important, as they form the foundation for the therapist’s responses.

In addition, the internet therapist must support the patient’s independence and self-management differently than in traditional therapy, guiding and motivating the patient while providing clear instructions for independent work. An interesting question, and a path for future research, is to identify how these elements can be best delivered in therapist-assisted iCBTs. In this study, patient accounts show that therapists could delve into these various ways of communication without face-to-face interactions. To further explore these elements, including factors such as reasons for dropout, new methods such as text mining can be used [eg, 38].

To further validate the relationships between the identified themes and adherence, a complementary quantitative or mixed methods approach could be considered for future research. For example, correlating the qualitative findings with quantitative measures, such as dropout rates or treatment completion, may provide a stronger basis for understanding the impact of these factors on therapy outcomes. This suggestion aligns with the findings by Mylläri et al [39], which revealed that machine learning techniques using text topics were effective in predicting dropout risk. By combining qualitative themes with such predictive models, future research could refine approaches to identifying patients at risk of dropout and developing strategies to improve adherence.

The findings of this study are important to consider in the future development of both self-guided and therapist-assisted iCBTs. The contributions outlined in this study provide valuable insights for health care practitioners, aiding in the design of patient-friendly internet therapies and enhancing the understanding of patient needs. For policy makers, these findings can serve as guidance in formulating policy recommendations for integrating technology into health care services, including addressing the imperative role of therapist involvement in delivering high-quality care.

### Strengths and Limitations

A strength of this study is the large number of interviews incorporating a variety of therapeutic programs (ie, different disorders). More precisely, the data represent a range of disorders, with the largest group being depression (32 cases). Furthermore, this study contributes to the discussion about the extent to which therapies can be automated and the role of the therapist in digital health care settings. In essence, it explores which elements can be effectively digitalized and which aspects must remain inherently human-driven.

This study did not consider user characteristics such as age or income. These unexamined factors, along with various others not explored in this study, may have influenced patients’ expectations, perceptions, and behavior. The focus of this study was on patients’ personal experiences, providing an in-depth understanding of their perspectives, but it does not offer a comprehensive view of all possible reasons for their adherence. In addition, this study does not differentiate between various iCBT programs, neglecting to observe potential differences between programs and disorders. Certain elements may be more critical in some programs than in others.

This study relies on retrospective answers, meaning patients discussed their past experiences. Recollection of experiences may introduce biases depending on the patients’ awareness of past events. In addition, the results hinge on patients’ willingness and ability to disclose information, and some may have been concerned that providing negative feedback could impact their future treatment, despite assurances in the interview information letter. As the data consisted of patients who volunteered to participate, they may have been highly motivated to join the research and may differ from other informants who did not want to join the interview.

One limitation of this study is the potential bias due to the first author’s central role in the analysis. This was addressed through systematic, collaborative coding, regular critical discussions with coauthors of complementary expertise, and a comprehensive audit trail documenting decisions and disagreements. The first author also engaged in recurrent self-reflection to bracket personal assumptions. While some bias is unavoidable in qualitative research, these measures enhanced rigor and validity. However, despite these measures to mitigate potential bias, some residual subjectivity cannot be entirely ruled out.

### Conclusions

This study aimed to explore patient perspectives on the key factors contributing to the effectiveness of iCBT, with a particular focus on the role of therapist support. Consistent with previous research on face-to-face therapy [27,28], elements that strengthen the therapeutic alliance and treatment adherence were found to be important in iCBT as well. In digital programs, patients’ sense of being seen, heard, validated, and supported often emerged from small, text-based interactions. This study highlights the pivotal role of therapist support, identifying 8 essential features that patients value—each of which is difficult to achieve without therapist involvement. The findings suggest that rather than replacing therapists with digital tools, integrating human and digital elements has the potential to significantly improve the effectiveness of iCBT.

## Abbreviations

HUS: Helsinki University Hospital
iCBT: internet-based cognitive behavioral therapy
OCD: obsessive-compulsive disorder
